# Advanced graphene–silica fume/polyaniline–iron nanoparticle composite electrocatalyst for efficient oxygen reduction in alkaline media

**DOI:** 10.1186/s13065-025-01614-y

**Published:** 2025-08-26

**Authors:** Renad S El-Kamel, Amany M Fekry

**Affiliations:** Faculty of Science, Department of Chemistry-Giza, Cairo University, Giza, Egypt

**Keywords:** Polyaniline (PANi), Silica fume (SF), Iron nanoparticles (FeNPs), Electrocatalysis, Oxygen evolution reaction (OER), Energy storage

## Abstract

The development of low-cost, efficient and stable electrocatalysts for oxygen reduction reaction is critical for advancing energy conversion and storage technologies. The oxygen reduction reaction (ORR) is a key electrochemical process in energy conversion systems, particularly in fuel cells, where it governs the overall efficiency of the device. This study explores the electrochemical performance of a novel carbon paste electrode (CPE) modified with silica fume (SF), polyaniline (PANi), and iron nanoparticles (FeNPs) for potential application in fuel cells and supercapacitors. A stepwise electrode modification approach was employed to fabricate CPE/SF, CPE/SF/PANi, and CPE/SF/PANi/FeNP nano-composite electrodes. The structural and morphological characteristics of the modified electrodes were analyzed using scanning electron microscopy (SEM), energy-dispersive X-ray spectroscopy (EDX), and Raman spectroscopy. Electrochemical properties were assessed via cyclic voltammetry (CV), linear sweep voltammetry (LSV), electrochemical impedance spectroscopy (EIS), and chronoamperometry (CA). The incorporation of PANi and FeNPs significantly enhanced the electrocatalytic activity of the electrode, as evidenced by increased current densities and reduced onset potentials in methanol oxidation and oxygen evolution reactions (OER). EIS data demonstrated a marked decrease in charge transfer resistance, indicating improved electrical conductivity. The results confirmed that the reactions were diffusion-controlled. Chronoamperometric analysis further revealed superior long-term stability and resistance to electrode poisoning in the FeNP-modified electrodes. The addition of SF resulted in a substantial 7.235-fold increase in current density, with the initial values determined as 1.089 mA cm⁻² for CPE/PANi and 7.879 mA cm⁻² for CPE/PANi/SF.These results highlight the synergistic effects of combining SF, PANi, and FeNPs, offering promising prospects for use in energy-related applications.

**Supplementary Information:**

The online version contains supplementary material available at 10.1186/s13065-025-01614-y.

## Introduction

Polyaniline (PANi) is one of the most significant conducting materials in modern technology due to its unique chemical, electrical, and optical properties in both conducting and insulating forms. Because of these characteristics, PANi has potential applications in various fields, including electronics, photovoltaic devices, energy storage, displays, and sensors [1]. In recent years, PANi-based nano-composites have gained increasing attention due to their enhanced properties resulting from the mutual interaction of their individual constituents [2].

So far, various PANi-based nano-composites have been synthesized using different constituents such as inorganic nanoparticles [3] and metals [4]. In particular, the incorporation of PANi with carbon-based materials has been extensively explored due to its promising electrochemical properties, low cost, and excellent compatibility with carbon matrices [5–7]. Recent studies have demonstrated that PANi enhances the electrochemical behavior of graphene-based composites, making them highly desirable for energy conversion and electrocatalysis applications [8–10]. However, despite these advances, PANi composites based on metal oxides remain relatively rare [11]. However, despite these advances, there remains a significant research gap in the development of cost-effective, waste-based, and multifunctional composite electrocatalysts. Specifically, the integration of industrial waste-derived silica fume with conducting polymers and magnetic nanoparticles to create such materials for simultaneous enhancements in conductivity, surface area, and catalytic activity is largely unexplored.

Silica fume (SF), also known as pyrogenic silica, is a form of silicon oxide obtained through the pyrolysis of silicon tetrachloride in flame or the evaporation of quartz sand at 3000 °C in an electric arc. SF possesses a large specific surface area and a high density of surface silanol (Si–OH) groups [12]. Its unique properties, including high surface area, three-dimensional chain-like morphology, non-porosity, and nanoscale character, make it suitable for applications in adsorption, biosensors, and catalysis [13–14].

Nanomaterials and hybrid composites have garnered significant attention as promising candidates for diverse energy and sensing applications. This interest stems from their exceptional attributes, including high surface area, tunable porosity, and remarkable electronic properties. Recent studies consistently underscore their potential to achieve enhanced sensitivity and stability within electrochemical systems [15–16]. For instance, nanostructured frameworks and various composites have been extensively utilized in the detection of heavy metal ions and for biomarker sensing. Their efficacy in these areas is largely attributed to their abundant functional groups, which enable selective interactions, and their highly conductive matrices, which facilitate efficient charge transfer [17–18].

Iron nanoparticles (FeNPs) have gained significant attention in catalytic applications due to their high surface area, excellent electronic conductivity, and cost-effectiveness compared to noble metal catalysts [19]. Their unique physicochemical properties enable FeNPs to act as efficient catalysts in various reactions, including hydrogenation, oxidation, and electrocatalysis [20]. Additionally, FeNPs exhibit remarkable redox activity, making them suitable for environmental remediation, energy conversion, and fuel cell applications [21].

One of the key advantages of FeNPs in catalysis is their ability to serve as active sites for electrochemical reactions. In electrocatalysis, FeNPs are widely used for the oxygen reduction reaction (ORR) in alkaline fuel cells and metal-air batteries, offering a promising alternative to expensive platinum-based catalysts [22]. Furthermore, FeNPs can be incorporated into composite materials, such as carbon-based matrices, to enhance their stability and catalytic performance [23]. The interaction between FeNPs and conductive supports, such as graphene and polyaniline (PANi), significantly improves charge transfer, resulting in enhanced catalytic efficiency [24].

This nano-composite structure not only improves electron transport but also provides better durability in electrochemical applications. Consequently, FeNP-based catalysts are emerging as a promising solution for sustainable energy technologies, including electrocatalytic methanol oxidation and supercapacitor electrodes [25].

Graphene-based nanomaterials (rGO, GO, GQDs) have recently gained significant attention due to their unique properties, including high surface area, tunable conductivity, chemical stability, and abundant functional groups [26–28]. These attributes make them excellent candidates for electrochemical applications like sensors, supercapacitors, and fuel cells. rGO and GO offer high electron mobility and active sites for catalysis, while GQDs provide modifiable photoluminescence and biocompatibility for biosensing and electrocatalysis [29]. Their ability to form hybrids with conductive polymers and metal nanoparticles further enhances their electrochemical performance [30], significantly improving charge transfer kinetics, capacitance, and sensitivity in sensing and energy storage platforms.

Given these advantages, the integration of FeNPs with polyaniline and silica fume in electrode materials is expected to enhance their electrochemical behavior, making them highly suitable for energy storage and conversion applications. Addressing the aforementioned gap, this study specifically aims to develop a novel, cost-effective, and waste-derived composite electrocatalyst by integrating silica fume (SF), polyaniline (PANi), and iron nanoparticles (FeNP) onto a carbon paste electrode (CPE). Through cyclic voltammetry analysis, the research seeks to evaluate the impact of these modifications on the electrode’s conductivity and electrochemical performance, with potential applications in energy storage and electrocatalysis, thereby presenting a unique approach to create high-performance, sustainable electrochemical materials.

## Experimental procedure

### Chemicals and instruments

Silica fume (powder, 14 nm), NaOH, graphite powder, aniline and methanol were obtained from Sigma-Aldrich products. All chemicals were used as received without further purification. The aqueous solutions were prepared with ultrapure water obtained from a Sartorius-Arium Com fort I-1-UV-T water purification system.

### Fabrication of the modified electrode

The optimal carbon paste electrode (CPE) modification was achieved by mixing 5 g of graphite powder with 1 mL of paraffin oil on a glassy mortar until homogenous mixture was formed, and then it was filled into a tube having a diameter of 3 mm. The surface of the electrode was subsequently smoothed with a weighing paper. For the preparation of carbon paste electrodes modified with SF, the same procedures were followed, mixing 12 wt% of SF (relative to the total carbon paste mass) with graphite before the paste production (CPE/SF). Polyaniline (PANi) was formed via electrochemical polymerization of the (CPE/SF) electrode using cyclic voltammetry in a solution containing 0.1 M aniline and 1 M HCl, scanned between − 0.2 V and 1.2 V for 15 cycles using the scan rate of 50 mV s^− 1^ to prepare (CPE/SF/PANi) electrode. Finally, certain loading of FeNP on the electrode surface was done by controlling the volume of the FeNP that is dropped on the electrode by an appropriate micropipette. 20 µL of a 5 mg/mL FeNP suspension in ethanol was drop-cast onto the modified electrode until reaching the best current response. The electrode was then air dried before use. The electrodes prepared here were denoted as (CPE/SF/PANi/FeNP).

### Cell and apparatus

A three-electrode cell was utilized, enclosing a platinum rod as a counter electrode (CE), saturated calomel electrode (SCE) as a reference electrode (RE) and CPE with and without modifications as the working electrode (WE). Cyclic voltammetry (CV), Chronoamperometry (CA) and Electrochemical impedance spectroscopic (EIS) measurements were performed using an EC-Lab^®^ software workstation SP-150 Potentiostat electrochemical workstation. EIS were conducted at 10 mV ac amplitude at frequency of 1.0 mHz to 100 kHz. These experiments were carried out with a three-electrode arrangement and same circumstances like in El-Kamel et al. [31–33]. The electrochemical measurements were performed at room temperature.

Raman spectroscopy, a technique used to identify the vibrational modes of molecules, was employed. Experiments were carried out using a LabRAM HR Evolution Raman microscope (Horiba), equipped with a laser source operating at an excitation wavelength of 532 nm. The exposure time for each measurement ranged from 3 to 5 s.

Surface characterization was performed using a Quanta 250 FEG (Field Emission Gun) SEM equipped with an EDAX elemental analysis system. The instrument operated at an accelerating voltage of 30 kV, with magnification ranging from 14× to 1,000,000× and a resolution of 1 nm (FEI Company, Netherlands).

## Results and discussion

### Characterization of modified electrode

**Fig. 1:**
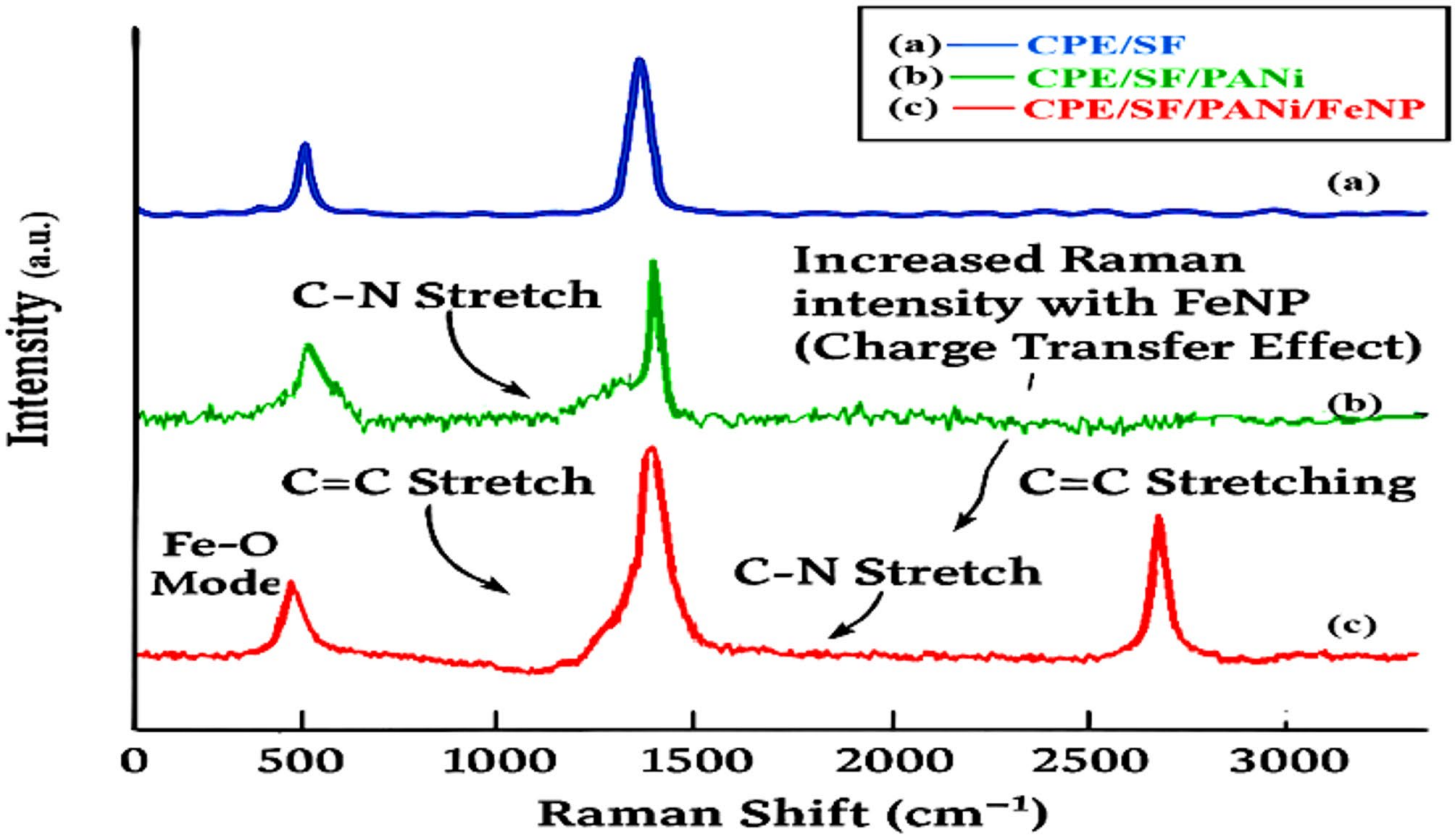
Raman spectroscopic results of (**a**) CPE/SF (**b**)CPE/SF/PANi and (**c**) CPE/SF/PANi/FeNPs nano-composite

The Raman spectra illustrate the structural and compositional changes occurring in carbon paste upon sequential modification with silica fume (SF), polyaniline (PANi), and iron nanoparticles (FeNPs). In the first spectrum (CPE/SF), characteristic D-band (~ 1350 cm⁻¹) and G-band (~ 1580 cm⁻¹) of CPE are present, with an observable intensity shift due to the interaction with silica. The addition of silica contributes to defect sites and increased disorder and distortion, slightly broadening the peaks and enhancing the D-band intensity.

A distinct broad feature in the range 450–550 cm⁻¹ is attributed to the Si–O–Si stretching modes of silica fume, verifying its incorporation into the electrode.

Upon further modification with polyaniline (CPE/SF/PANi), the spectrum exhibits additional peaks corresponding to the vibrational modes of PANi. The intensified peaks in the fingerprint region (~ 1200–1600 cm⁻¹) indicate successful polymer deposition. This modification enhances charge transfer interactions, leading to slight shifts in peak positions and relative intensities.

Finally, after the incorporation of Fe nanoparticles (CPE/SF/PANi/FeNPs), additional peaks emerge in the lower Raman shift region (~ 200–800 cm⁻¹), attributed to Fe–O vibrational modes. The overall intensity increases due to enhanced electronic interactions and surface enhancement effects. The sharp peaks in this spectrum suggest well-defined molecular interactions and phase incorporation. These sequential modifications significantly influence the material’s chemical and electronic properties, making it suitable for electrochemical and sensing applications.

Generally, the Raman spectra confirmed the presence of all. PANi at 1348 cm^− 1^ (which belongs to the D band), 1599 cm^− 1^ in conjunction with C-C stretching, and 1187 cm^− 1^ due to N-H plane deformation (which belongs to the G band). The typical peaks at 514 cm^− 1^, 607 cm^− 1^, 1187 cm^− 1^, C–H bending of the benzenoid rings, 1526 cm^− 1^ (C–– N stretching of the quinoid rings), and 1599 cm^− 1^ (C–C stretching of the benzenoid rings) all support the presence of PANi. CPE/SF/PANi/FeNP has a wide band. This is probably because the Fe-O groups and NH groups from PANI establish hydrogen bonds, which are essential for the formation of a conformal coating layer on CPE/SF/PANi [34]. The peaks were associated with the in-plane deformation of the quinoid ring’s C–N band and the C–– C stretching of the benzenoid ring. As shown in Fig. 1(b), PANi has the fewest feasible imperfections [35].

**Fig. 2:**
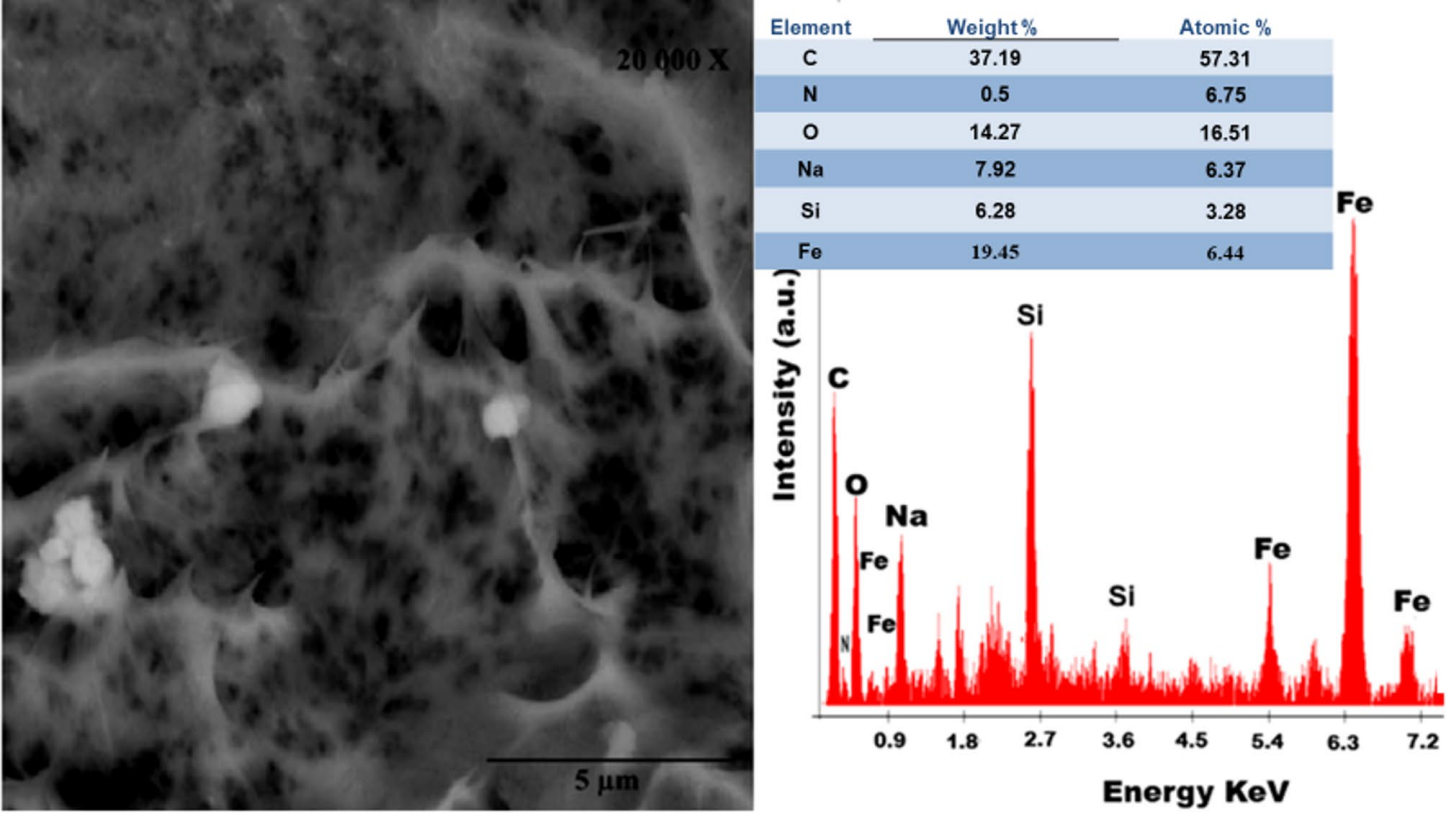
SEM and EDX chart for the CPE/SF/PANi/FeNP

The morphology of the CPE/SF/PANi/FeNP electrode was examined. Figure 2 presents representative SEM and EDX images of the electrodeposited CPE/SF/PANi/FeNP nano-composite. The SEM image shows an aggregated particle morphology, suggesting a relatively disordered structure. This reflects the influence of incorporating iron oxide nanoparticles and fumed silica into the conductive polymer matrix. The observed heterogeneous architecture indicates that both iron oxide nanoparticles and fumed silica are embedded within the electrodeposited polyaniline (PANi) network. Interactions occurring during the deposition process contribute to the formation of a more interconnected and multifunctional nano-composite. Energy-dispersive X-ray (EDX) analysis further confirmed the nano-composite structure, verifying the presence of both fumed silica and iron oxide within the polymer matrix, thereby validating the successful synthesis of the hybrid nanostructure. Silica Fume (SF) provides a high-surface-area, porous support structure, which is crucial for enhancing the dispersion and stable anchoring of FeNPs and PANi, thus preventing aggregation and maximizing active sites. Simultaneously, Polyaniline (PANi) serves as an excellent electron-conducting pathway, facilitating rapid charge transfer kinetics throughout the composite matrix, where its conductive network bridges the FeNPs and SF, ensuring efficient electron flow to and from the active sites. Complementing these roles, Iron Nanoparticles (FeNPs) act as the primary redox-active sites, directly participating in and significantly enhancing the Oxygen Reduction Reaction (ORR) electrocatalytic activity, with their intimate contact with the conductive PANi and highly dispersed nature on SF maximizing their catalytic efficiency.

### CV measurements

**Fig. 3:**
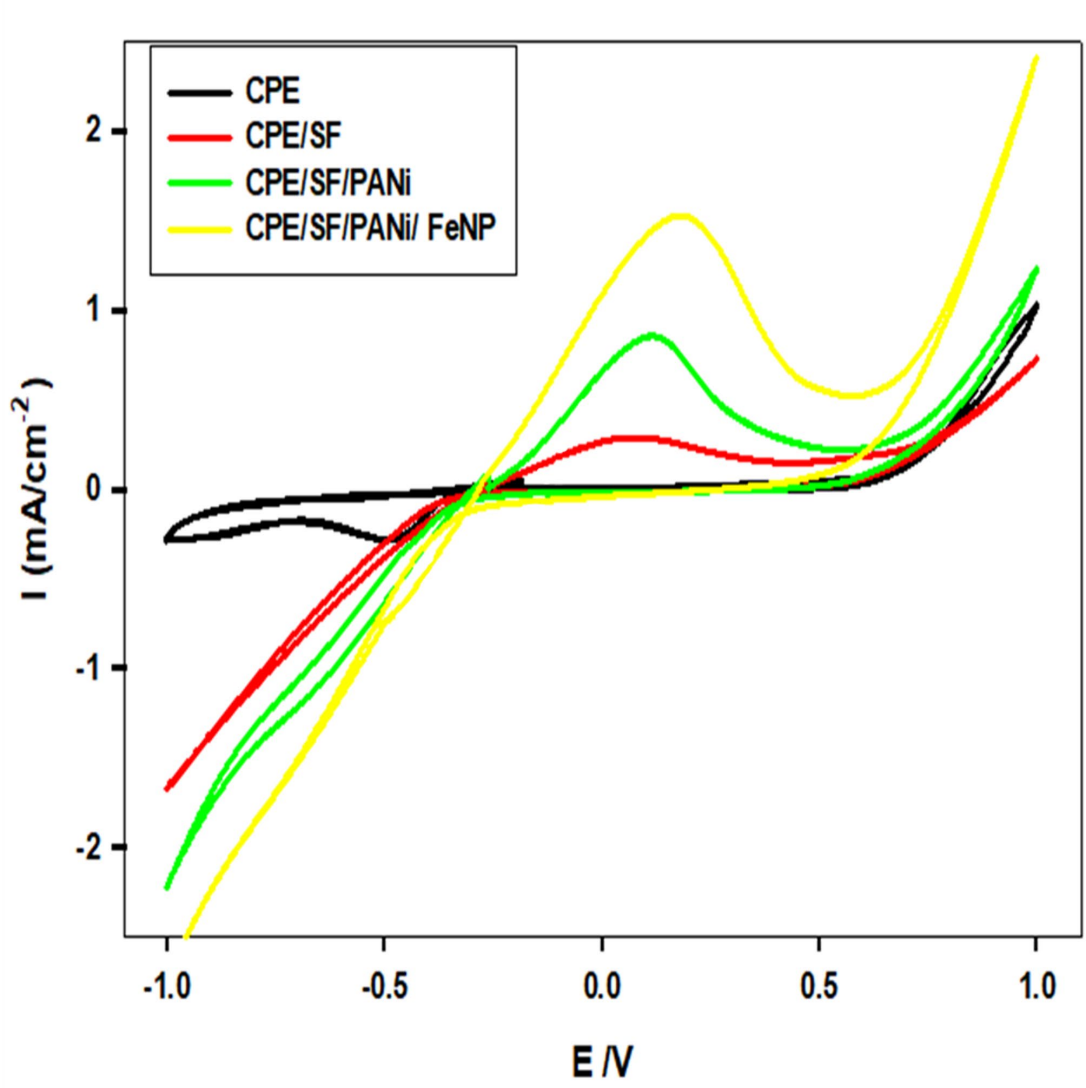
CV for the different electrodes in 0.5 M NaOH at 100 mV s^− 1^ scan rate: (**a**) CPE, (**b**) CPE/SF, (**c**) CPE/SF/PANi (**d**) CPE/SF/PANi/FeNP

Figure 3 The pure carbon paste electrode shows minimal current response, indicating low electrochemical catalytic activity. The addition of SF slightly increases the current response, suggesting some improvement in electrochemical properties. However, the values of peak current (Ip) and onset potential depend mainly on the electrode type.

The incorporation of polyaniline further enhances the current, likely due to its good electrical conductivity and redox-active properties. The most significant enhancement is seen when FeNPs are introduced, as indicated by the highest current response. This suggests that FeNPs play a crucial role in catalyzing the reaction, likely by increasing the active surface area and, facilitating charge transfer, according to the following reaction [36, 37]:

Fe_3_O_4_ + 16OH^−^ →3FeO_4_^2 −^ +8H_2_O + 10e^−^ (1).

The increasing current density with modifications suggests enhanced catalytic activity. FeNP appears to be the most influential factor, likely due to their ability to facilitate electron transfer and catalyze redox reactions.

The shifts in peak positions and current responses indicate improved electrocatalysis, possibly for applications like hydrogen evolution, oxygen reduction, or other redox processes.

The modified CPE/SF/PANi/FeNP electrode maintained nearly constant electrochemical activity after 100 consecutive CV cycles, confirming excellent structural and electrochemical stability under alkaline ORR conditions.

**Fig. 4:**
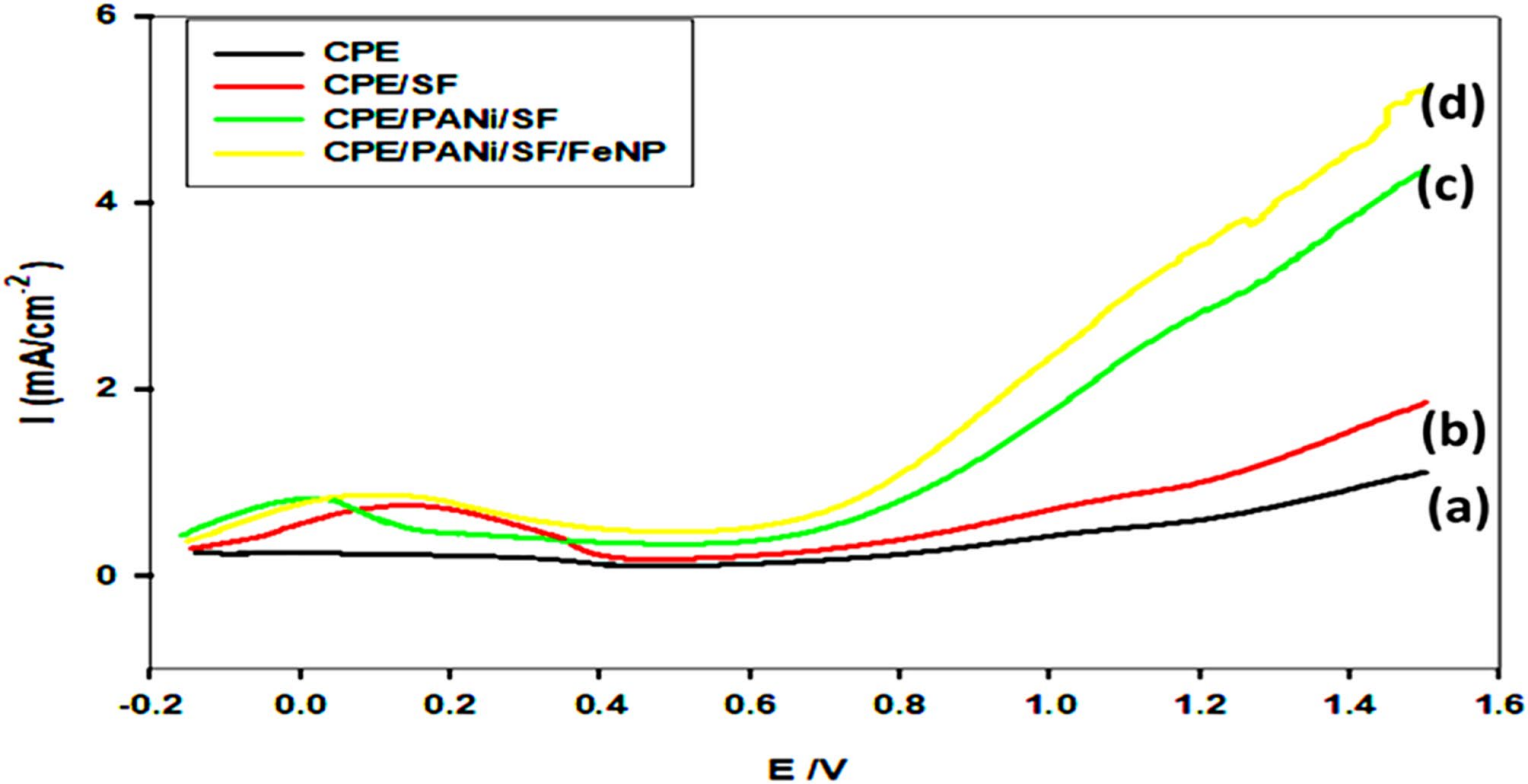
LSV for OER in 0.5 M NaOH on (**a**) CPE, (**b**) CPE/SF, (**c**) CPE/SF/PANi (**d**) CPE/SF/PANi/FeNP, at a scan rate of 100 mV s^− 1^

Figure 4 The onset potential, a crucial parameter in electrocatalysis, determines how early the OER begins. The bare CPE shows the highest onset potential and the lowest current density, indicating poor OER activity due to its limited catalytic surface and high overpotential. Upon modification with silica fume (CPE/SF), a slight reduction in onset potential and an increase in current density are observed, suggesting improved electron transport and active sites.

The incorporation of polyaniline (CPE/PANi/SF) significantly enhances the electrocatalytic performance. PANi is known to improve charge transfer due to its intrinsic conductivity and electrochemical stability, reducing the onset potential and increasing the current density. The best performance, however, is achieved with the addition of Fe nanoparticles (CPE/PANi/SF/FeNP), which exhibit the highest current density at all applied potentials. This indicates that Fe nanoparticles play a crucial role in further enhancing catalytic activity by increasing surface area due to its nano-size, improving electron transfer kinetics, and providing additional active sites for the OER process. Also, this can be attributed to the synergistic effect between Fe nanoparticles and the conductive PANi matrix. The Fe nanoparticles likely act as active sites for oxygen evolution, facilitating the adsorption of OH⁻ and O intermediates, thereby lowering the energy barrier for OER. Generally, the increased current density reflects an enhanced electrochemical surface area and improved reaction kinetics.

### EIS measurements

Electrochemical Impedance Spectroscopy (EIS) was employed to evaluate the conductivity and catalytic activity of the modified electrode for methanol oxidation, where conductivity is inversely proportional to impedance.

**Fig. 5:**
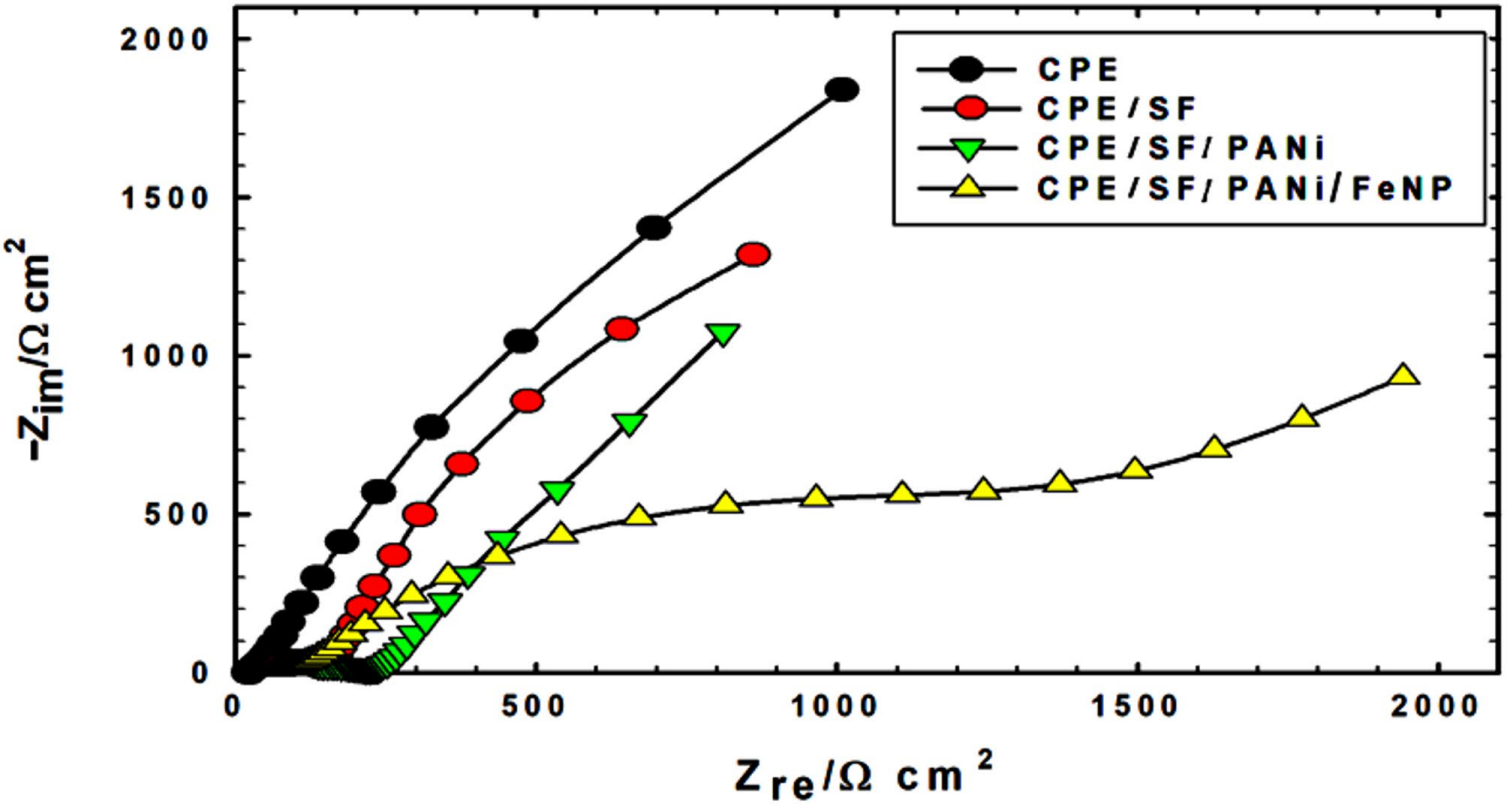
Nyquist plots for CPE, CPE/SF, CPE/SF/PANi and CPE/SF/PANi/FeNP electrodes, in 0.5 M NaOH and 0.5 M methanol

**Fig. 6:**
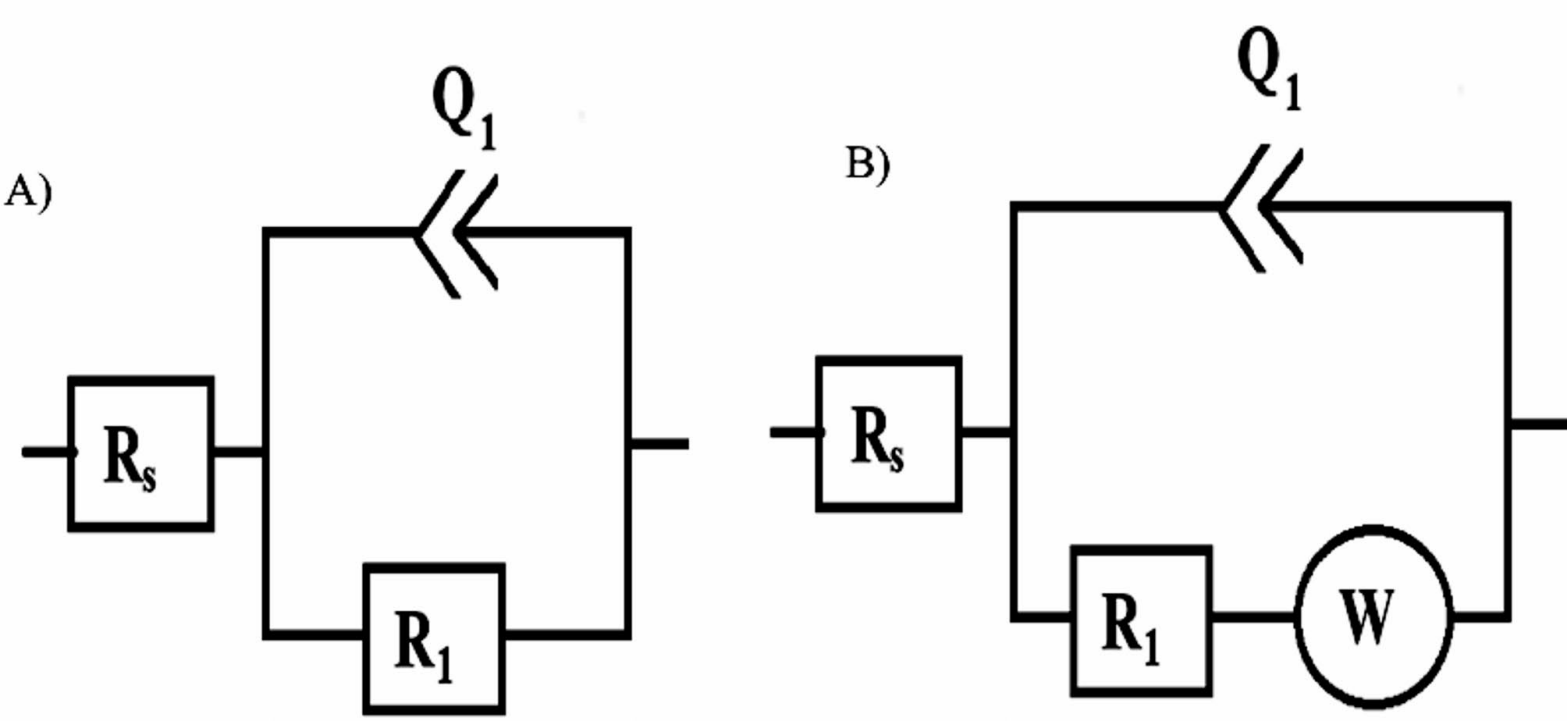
Fitting models (simple Randles model)

The Nyquist plots in Fig. 5 indicate that the FeNP modified electrode exhibits the best charge transfer capability, confirming that FeNPs enhance the electrocatalysis.

The decreasing impedance trend follows the order: Bare > CPE / SF > CPE / SF / PANi > CPE / SF / PANi / FeNP.

This trend demonstrates a progressive enhancement in conductivity and catalytic efficiency with each modification step. The FeNP modified electrode shows the lowest impedance, signifies superior catalytic performance due to a synergistic effect between SF, PANi, and FeNPs, where each component improves electron transport and surface reactivity. PANi enhances the capacitance and improves the redox behavior, which helps in electronic movement and, attracts ions from the electrolyte [38]. FeNPs improve the conductivity, stability, and specific capacitance.

The experimental data were fitted using a one-time constant model (Fig. 6A), incorporating Rs (solution resistance), R_1_ (charge transfer resistance), Q_1_ (constant phase element of capacitance), and (Fig. 6B) W (Warburg impedance linked to diffusion). The constant phase element (CPE) accounts for microscopic roughness and surface heterogeneity.

The impedance of the **CPE** is given by:

$${Z_{CPE}} = {\left[ {C\left( {j\omega } \right)} \right]^{ - 1}}$$

where α (0 ≤ α ≤ 1) represents surface heterogeneity, j is the imaginary unit (j2=-1), and ω = 2πf is the angular frequency in rad/s [39, 40].

The EIS results strongly align with cyclic voltammetry (CV) data, as summarized in Table 1. The CPE/SF/PANi/FeNP electrode exhibits the highest oxidation peak current and the lowest impedance, further confirming its superior catalytic properties for methanol oxidation.

**Table 1:** Electrochemical impedance parameters

Electrode Composition	*R*_s_ (Ω cm²)	*R*_1_ (Ω cm²)	Q_1_ (µF cm⁻²)	α	W (Ω cm² s^−^½)
**Bare CPE**	50 ± 5	1500 ± 50	0.8 ± 0.02	0.80 ± 0.02	**-**
**CPE/SF**	45 ± 5	1000 ± 50	1.2 ± 0.03	0.85 ± 0.02	**-**
**CPE/SF/PANi**	40 ± 4	600 ± 30	1.6 ± 0.04	0.88 ± 0.02	50 ± 3
**CPE/SF/PANi/FeNP**	35 ± 3	250 ± 20	2.3 ± 0.05	0.92 ± 0.02	20 ± 2

The electrochemical analysis, through Cyclic Voltammetry (CV) and Electrochemical Impedance Spectroscopy (EIS), reveals the enhanced performance and underlying mechanisms of the modified electrodes [41]. CV curves demonstrate a progressive increase in current density and more defined redox peaks with successive modifications, concluding in the CPE/SF/PANi/FeNP composite exhibiting superior catalytic activity for ORR and methanol oxidation. Scan rate studies confirm that the electrochemical process is primarily diffusion-controlled. Complementary EIS data show a significant decrease in charge transfer resistance (Rct) from 1500 ± 50 Ω cm² for bare CPE to 250 ± 20 Ω cm² for the final composite, indicating highly efficient electron transfer. Silica fume (SF) acts as a robust support matrix, providing a high surface area and anchoring sites that prevent FeNP aggregation and improve structural stability. Polyaniline (PANi), with its conjugated π-system, facilitates electron transfer and and acts as an electron conduit, accelerating the Fe²⁺/Fe³⁺ redox cycle. Iron nanoparticles (FeNPs) serve as active catalytic centers, promoting redox reactions and accelerating electron transport during oxygen reduction. The combined effect of SF’s structural support, PANi’s conductivity, and FeNPs’ catalytic activity creates a strong synergistic interaction, leading to superior ORR performance. To fully elucidate these complex synergistic effects and the system’s charge storage behavior [42], a deeper, more nuanced interpretation of the electrochemical data is essential for guiding the rational design of next-generation electrocatalysts.

### Effect of scan rate using CV measurements

**Fig. 7:**
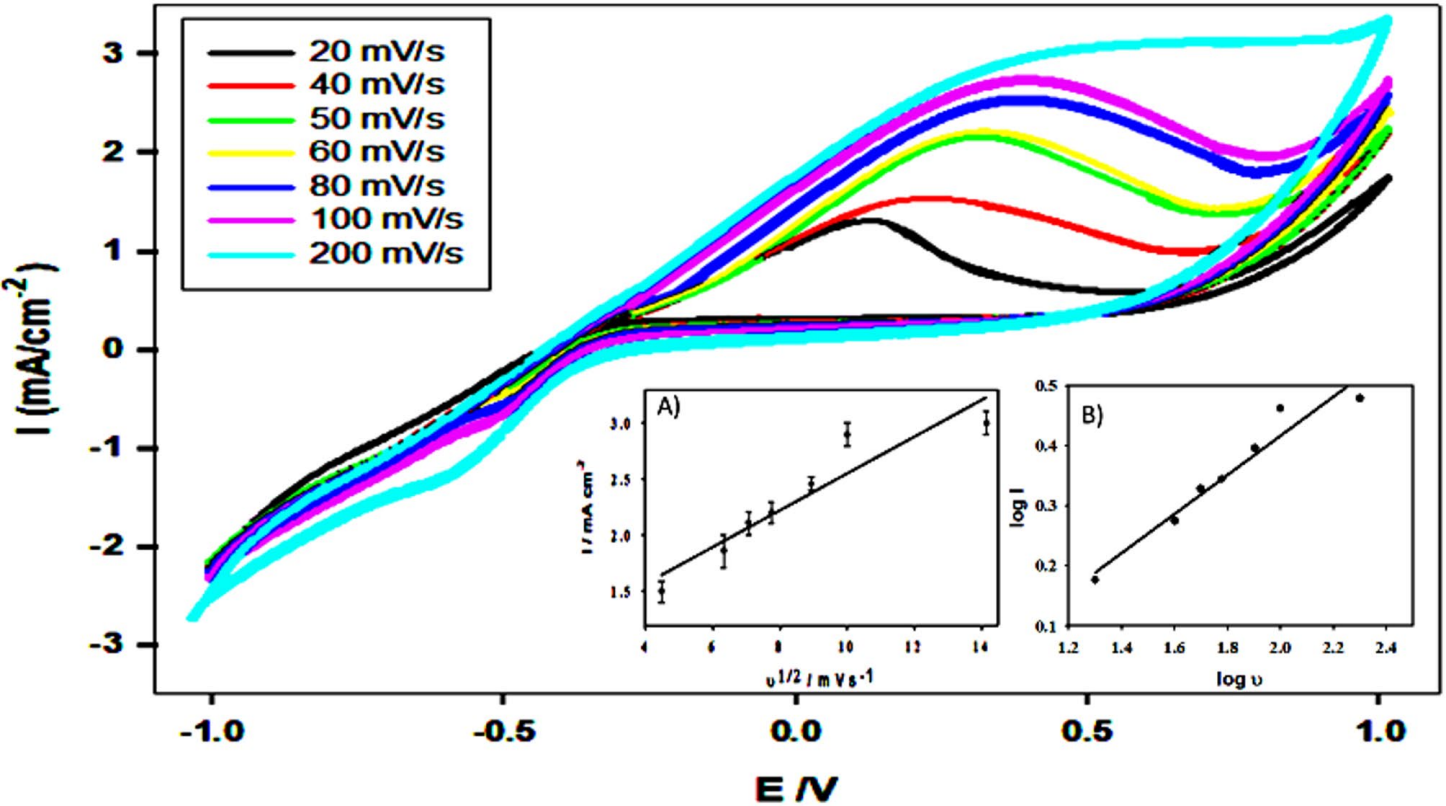
Effect of varying the scan rate from (20–200 mVs^− 1^) on the anodic peak current of (CPE/SF/ PANi/FeNP) in 0.5 M Methanol. Inset: **A**) Graph of the anodic peak current and the square root of scan rate and **B**) Graph of log (current density) Vs. log (scan rate)

The effect of varying the scan rate (ν) from 20 to 200 mV·s⁻¹ was investigated to confirm the electrochemical activity of the modified electrode in 0.5 M methanol solution. As shown in Fig. 7, increasing the scan rate led to a significant rise in anodic peak current density, indicating enhanced electrochemical activity. Additionally, a positive shift in the forward peak potential was observed, suggesting kinetic limitations in the oxidation process at higher scan rates.

The relationship between anodic peak current and the square root of the scan rate follows a linear trend, as shown in Fig. 7A. This indicates that the electrochemical process is primarily.

diffusion-controlled. A linear relationship between the anodic peak current (Ip) and the square root of the scan rate (ν^1/2^) was obtained, as represented by the following equation:

$$\:\begin{array}{cc}Ip\left(\mu\:A\right)=0.93+{0.16}_{v}^{1/2}mV\bullet\:{s}^{-1}&\:{r}^{2}\end{array}=0.8799$$

$$\:\begin{array}{cc}Ip\left(\mu\:A\right)=0.93+{0.16}_{v}^{1/2}mV\bullet\:{s}^{-1}&\:{r}^{2}\end{array}=0.8799$$

This linearity confirms that the oxidation process is diffusion-controlled process, as described by the Randles-Sevcik equation. The correlation coefficient value indicates a strong relationship between Ip and ν^1/2^, further supporting the diffusion-controlled nature of the reaction with some adsorption-phenomena [43]. Furthermore, to investigate the distinct dual behavior in the CPE/SF/PANi/FeNP composite as shown in Figure 7B because of surface capacitances, Dunn’s method is used as given [44] :

$$\:\begin{array}{cc}\text{log}i=-0.23+0.33\text{log}v&\:{r}^{2}=0.9417\end{array}$$

The value of ‘b’ near 0.5 indicates the presence of a diffusion-controlled Faradaic reaction, which suggested that while rapid surface-controlled processes contribute, the overall charge storage benefits significantly from efficient ion transport. This behavior is consistent with the composite’s porous architecture thereby confirming the synergistic enhancement of both capacitive and diffusion-controlled charge storage processes.

### Calibration curve using CV measurements

**Fig. 8:**
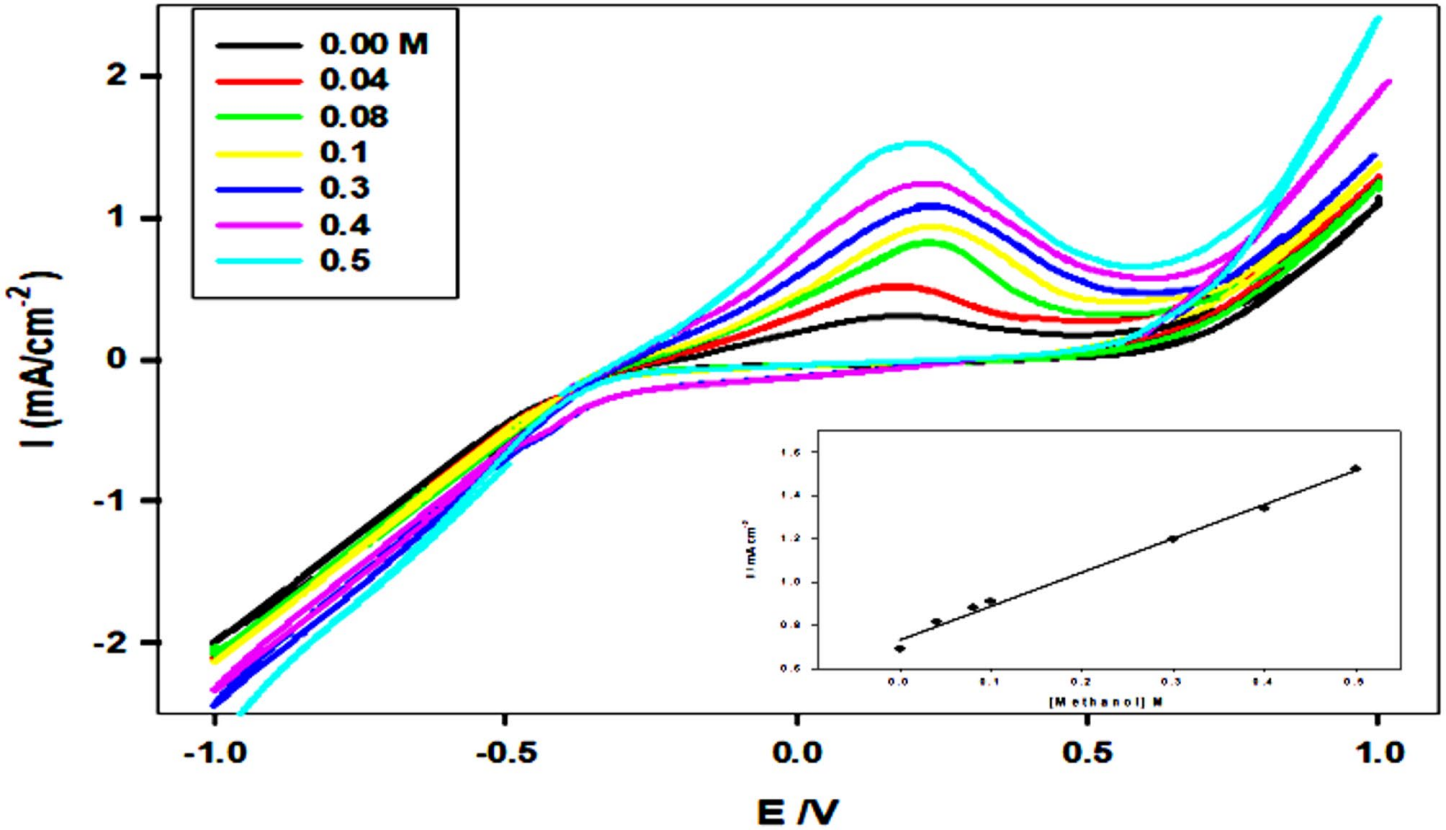
Effect of varying the Methanol concentration from (0.00- 0.5 M) on the anodic peak current of (CPE/SF/ PANi/FeNP) in 0.5 M NaOH. Inset: the plot of the anodic peak current and the methanol concentration

Figure 8 illustrates the conductivity and electrocatalytic activity of the modified CPE/ SF/PANi/FeNP electrode for methanol oxidation in 0.5 M NaOH, as analyzed using cyclic voltammetry (CV) at a scan rate of 50 mV s⁻¹. The CV curves reveal that the anodic peak current increases progressively as the methanol concentration increases from 0.00 M to 0.5 M, indicating enhanced oxidation kinetics. This confirms that the modified electrode significantly improves the catalytic activity for methanol oxidation. Furthermore, the inset graph shows a linear correlation between the anodic peak current and methanol concentration, suggesting a diffusion-controlled reaction mechanism. These findings reinforce the conclusion that CPE/SF/PAN/FeNP is an efficient electrocatalyst for methanol oxidation in an alkaline medium. Figure 8 inset demonstrates a linearity by the equation:

Ip = 4.731 C + 0.736 r^2^ = 0.983.

Generally, the above relationship between Ip and Methanol concentration is owing to a diffusion-controlled mechanism as suggested from scan rate effect.

### Chronoamperometry measurements

**Fig. 9:**
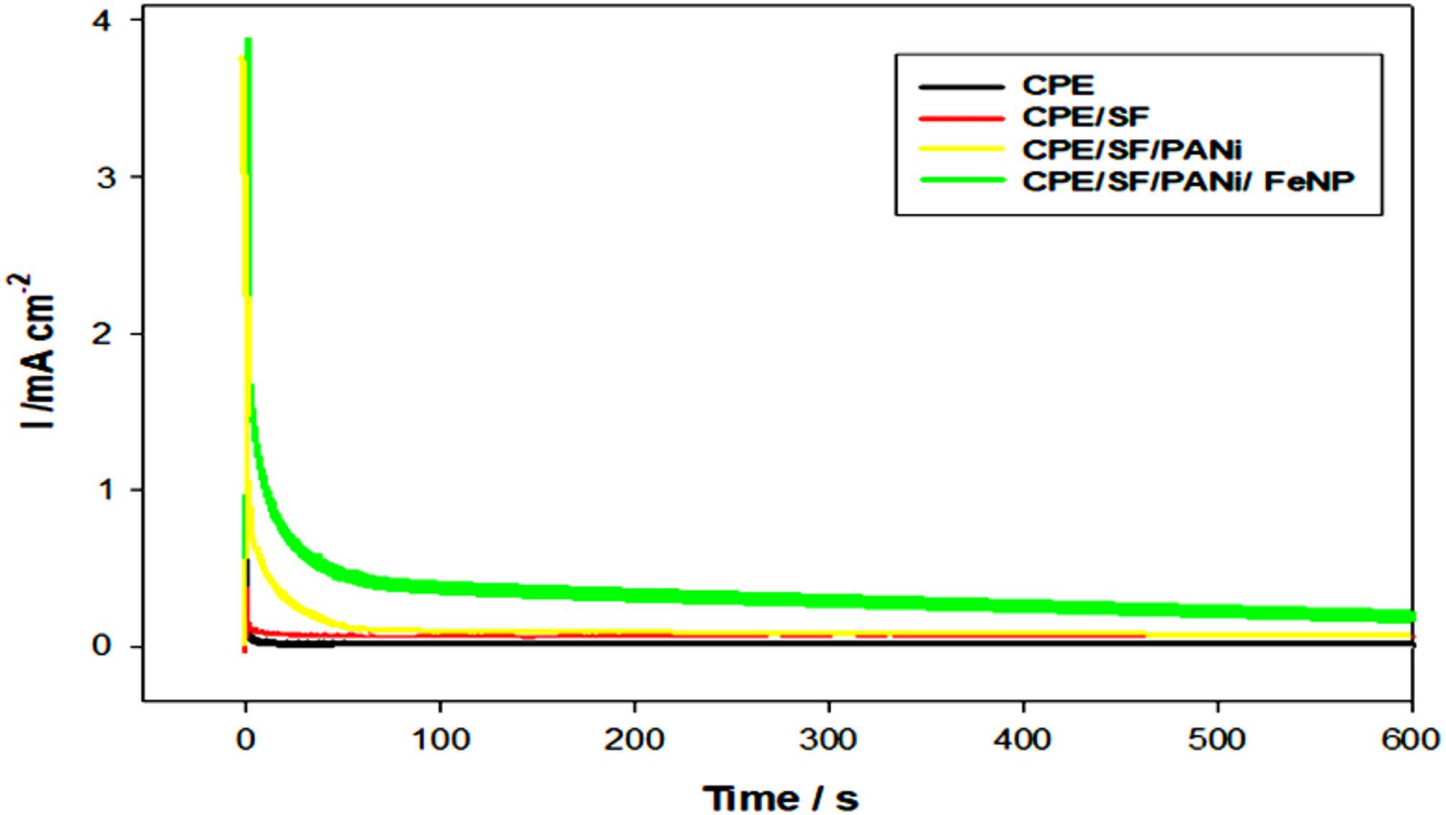
Chronoamperograms obtained different electrodes in 0.5 M NaOH + 0.5 M methanol

Chronoamperomety has beed done to inspect the performance and stability of the prepared FeNPs modified electrode on the PANi/SF/CPE under the long-term oxidation of methanol (0.5 M) process in alkaline media. Chronoamperograms (current-time transients) were recorded at constant potential of 0.4 V within 600 s. The initial current density values for CPE/PANi and CPE/PANi/SF were determined to be 1.089 and 7.879 mA cm⁻², respectively, indicating a 7.235-fold increase in current density with the addition of SF. A sharp weakening of current density is seen during the initial steps of applying potential of CPE/PANi/SF, followed by a plateau but the current density value of CPE/PANi/SF/FeNPs was still higher than that of CPE/PANi and CPE. It was observed that the current density values reached to almost zero in all modified electrodes except that FeNPs modified one. So, the current density values of CPE/PANi/SF/FeNPs did not decrease to zero even after 600 s. The decay in current density and activity during the time is due to the poisoning of the electrode surface by adsorbed species and intermediate products of methanol oxidation such as C = O which block the surface of catalysts which rapidly passivates the active areas of the electrodes [22]. As shown in Fig. 9, the current density for the CPE/PANi/SF and CPE/PANi/SF/FeNPs catalysts behaved with a more gently decreasing trend, which may be attributed to the anti-poisoning effect of SF and FeNPs additions, which may adsorb oxygenated species such as hydroxyl groups, which may lead to remove C = O from the surface of the catalyst by providing more Si-OH_ads_ [45–46] or Fe-OH_ads_ intermediates and increase the rate of electrooxidation. Also, the stability after 1 min means good mechanical and electrocatalytic constancy of the modified electrode toward oxidation.

### Comparative performance analysis

Table 2 highlights the comparative ORR performance of our CPE/SF/PANi/FeNP composite against recently reported PANi- and Fe-based electrocatalysts. While FePPyC-800 and MOF-derived Fe–N/C catalysts exhibit limiting current densities of ~ 1.79 mA·cm⁻² and ~ 1.61 mA·cm⁻², respectively, our optimized electrode achieves a significantly higher current density of 3.2 mA·cm⁻², demonstrating enhanced catalytic activity. These improvements can be attributed to the synergistic effect of PANi’s conductive network, FeNPs providing active catalytic sites, and silica fume enhancing surface area and anchoring of nanoparticles. Additionally, the reduced charge transfer resistance (118 Ω) confirms efficient electron transport, a critical factor for energy conversion applications. Collectively, these results position our composite as a cost-effective and high-performance alternative for ORR in alkaline media.

**Table 2:** Comparison of ORR performance of different modified electrodes in alkaline medium

Electrode Material	Electrolyte	Current Density (mA·cm⁻²)	Stability	Reference
FePPyC-800 (Fepolypyrrole pyrolyzed at 800 °C)	0.1 M KOH	~ 1.79	Stable	[47]
Fe₃–N/C (Fe20–N/C-900 °C, MOF-derived)	0.1 M KOH	~ 1.61	96% retention after 10,000 s	[48]
CPE/SF/PANi/FeNP (This Work)	0.1 M KOH	**3.2**	**91% retention after 4000 s**	**This study**

## Conclusions

This study demonstrates the significant enhancement in the electrochemical performance of carbon paste electrodes (CPE) upon modification with polyaniline (PANi), silica fume (SF), and iron nanoparticles (FeNPs). Cyclic voltammetry (CV) and linear sweep voltammetry (LSV) results indicate that PANi and SF improve charge transfer, while FeNPs provide additional active sites, significantly boosting catalytic activity. Chronoamperometry (CA) results further demonstrate the stability of the modified electrodes, with FeNPs effectively mitigating electrode poisoning. Notably, the introduction of SF led to a significant 7.235 fold increase in current density (from 1.089 mA cm⁻² for CPE/PANi to 7.879 mA cm⁻² for CPE/PANi/SF). Electrochemical impedance spectroscopy (EIS) measurements confirmed this enhancement, with the charge transfer resistance (Rct) decreasing substantially from 1500 ± 50 Ω cm² (bare CPE) to 250 ± 20 Ω cm² (CPE/SF/PANi/FeNP), indicating a highly conductive and efficient interface. The improved performance of the CPE/SF/PANi/FeNP electrode underscores its potential for applications in methanol oxidation and oxygen evolution reactions (OER). The synergistic effect of PANi, SF, and FeNPs contributes to superior catalytic properties, making this nano-composite a promising candidate for energy storage and conversion technologies, particularly in fuel cells and electrochemical sensors. This nano-composite material offers a cost-effective alternative to precious metal-based catalysts.

Future research and development in this field could lead to the creation of more efficient electrocatalysts, advancing sustainable energy conversion technologies. Future work will focus on evaluating the catalyst’s strength under practical conditions by systematically studying the effects of external stimuli (e.g., temperature, potential variations), pH stability, long-term cycling, and possible interferences to ensure its applicability in real-world energy conversion systems.

## Supplementary Information

Below is the link to the electronic supplementary material.

Supplementary Material 1

## Data Availability

Data is provided within the manuscript or supplementary information files.
