# Supplementary figures and images for: Advanced graphene–silica fume/polyaniline–iron nanoparticle composite electrocatalyst for efficient oxygen reduction in alkaline media
Source: BMC Chem. 2025 Aug 26;19(1):251. doi: 10.1186/s13065-025-01614-y (PMC12382175; doi:10.1186/s13065-025-01614-y)

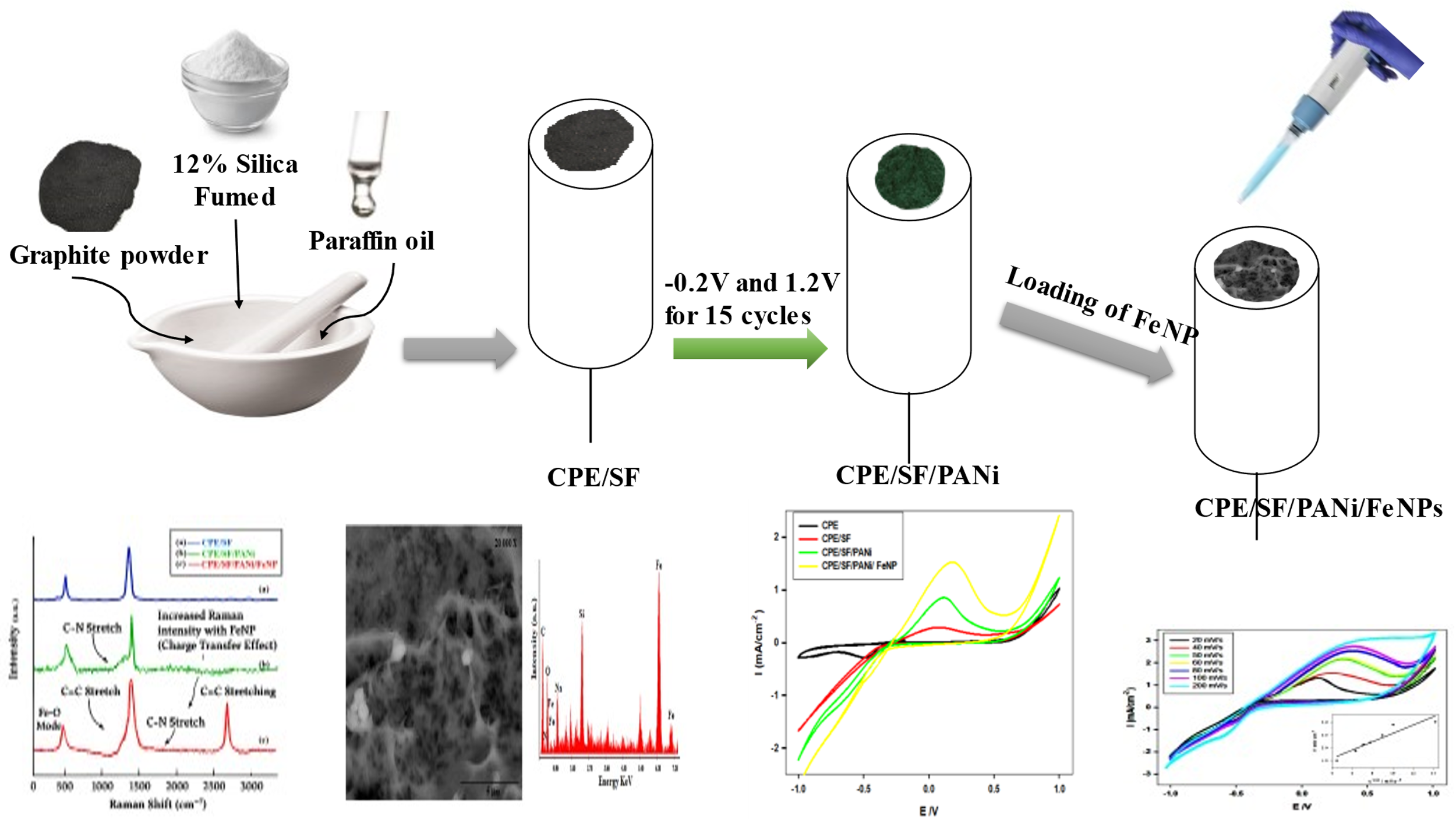

Supplement: Supplementary file 1 — Supplementary Material 1 [file 13065_2025_1614_MOESM1_ESM.docx]
